# 4026 Dissecting the role of microenvironment heterogeneity on metastatic tumor cell phenotype at an engineered metastatic niche

**DOI:** 10.1017/cts.2020.62

**Published:** 2020-07-29

**Authors:** Sophia Orbach, Michael D. Brooks, Grace G. Bushnell, Max S. Wicha, Jacqueline S. Jeruss, Lonnie D. Shea

**Affiliations:** 1 University of Michigan School of Medicine; 2 University of Michigan

## Abstract

OBJECTIVES/GOALS: Breast cancer metastases are stochastic and difficult to detect. Therapy is often ineffective due to phenotypic changes of tumor cells at these sites. We engineered a synthetic metastatic niche to study the role of phenotypic transitions in the microenvironment on tumor cell phenotype. METHODS/STUDY POPULATION: The engineered metastatic niche is composed of a porous polycaprolactone scaffold implanted subcutaneously in Balb/c mice. The mice received an orthotopic inoculation of 4T1 cells (murine triple negative breast cancer) in the fourth right mammary fat pad and the disease was allowed to progress for 7-21 days (pre-metastatic to overt metastatic disease). The scaffolds and lungs (native metastatic site) were explanted and analyzed by single cell RNA-seq via Drop-seq. Cell phenotypes were identified and tracked over time with the Seurat and Monocle3 pipelines. Assessment of the impact of these cell populations on tumor cell phenotype was conducted through Transwell co-cultures. RESULTS/ANTICIPATED RESULTS: Healthy scaffolds are primarily composed of macrophages, dendritic cells, and fibroblasts – consistent with a foreign body response. Despite differences in the lung and scaffold prior to tumor inoculation, both tissues were marked by >5-fold increase in neutrophils/MDSCs. Additionally, 79% of genes at the scaffold that significantly changed over time were also identified in the lung, indicating key similarities in niche maturation. However, many immune cells at the scaffold had distinct phenotypes, with pro-inflammatory/cytotoxic characteristics. These changes clearly impacted tumor cell phenotype, as cells from the scaffold increased tumor cell migration and apoptosis *in vitro*. DISCUSSION/SIGNIFICANCE OF IMPACT: Early phenotypic changes at the engineered metastatic niche can identify signs of metastasis prior to colonization of tumor cells. Furthermore, dynamics of immune and stromal cells change throughout niche maturation, influencing tumor cell phenotype and may suggest targeted therapies. CONFLICT OF INTEREST DESCRIPTION: Lonnie Shea, Jacqueline Jeruss, and Grace Bushnell are named inventors on patents or patent applications.

